# Hematological and hepatic alterations in nonsmoking residents exposed to benzene following a flaring incident at the British petroleum plant in Texas City

**DOI:** 10.1186/1476-069X-13-115

**Published:** 2014-12-20

**Authors:** Mark A D’Andrea, G Kesava Reddy

**Affiliations:** University Cancer and Diagnostic Centers, 12811 Beamer Road, Houston, 77089 TX USA

**Keywords:** Benzene poisoning, Blood disorders, Chemical exposure, Health impact, Hematological toxicity, Hepatotoxicity, Nonsmoking, Petroleum refinery

## Abstract

**Objective:**

Human exposure to benzene is associated with multiple adverse health effects with an increased risk of developing carcinogenesis. Benzene exposure is known to affect many critical organs including the hematological, hepatic, renal, lung, and cardiac functions. The purpose of this study is to examine the health effects of benzene exposure among nonsmoking subjects from a prolonged flaring incident that occurred at the British petroleum (BP) refinery in the Texas City, Texas.

**Methods:**

The study included nonsmoking subjects who had been exposed and unexposed to benzene. Using medical charts, clinical data including white blood cell (WBC) counts, platelet counts, hemoglobin, hematocrit, blood urea nitrogen (BUN), creatinine, alkaline phosphatase (ALP), aspartate amino transferase (AST), and alanine amino transferase (ALT) in nonsmoking subjects exposed to benzene were reviewed and analyzed and compared with unexposed adults.

**Results:**

A total of 1422 nonsmoking subjects (benzene exposed, n = 1093 and unexposed, n = 329) were included. Benzene exposed subjects had significantly higher levels of WBC (× 10^3^ per μL) counts (7.7 ± 2.2 versus 6.8 ± 1.7, P = 0.001) and platelet (× 10^3^ per μL) counts (288.8 ± 59.0 versus 245.3 ± 54.4, P = 0.001) compared with the unexposed subjects. The mean serum creatinine (mg/dL) levels were also significantly increased in the benzene exposed group compared with the unexposed group (1.1 ± 0.4 versus 0.8 ± 0.2, P = 0.001). Serum levels of ALP (IU/L) was significantly elevated in the benzene exposed subjects compared with the unexposed subjects (87.3 ± 22.6 versus 69.6 ± 16.5, P = 0.001). Similarly, benzene exposed subjects had significantly higher levels of AST and ALT compared with those unexposed subjects.

**Conclusion:**

Benzene exposure from the prolonged BP flaring incident caused significant alterations in hematological and liver markers indicating that these nonsmoking residents exposed to refinery chemicals may be at a higher risk of developing hepatic or blood related disorders.

## Introduction

Benzene is a major constituent of petroleum and occurs naturally in crude oil. It is also formed as a result of the incomplete combustion of fossil fuels such as petroleum products and coal. In addition, benzene is a commercially important intermediate in the manufacture of many chemicals [1–4]. Thus, petroleum refining industries are the major sources of benzene and other toxic chemicals. Benzene is one of the most widely used chemicals in the synthesis of various polymers, resins, and synthetic fibers. Moreover, benzene is a common component of gasoline [5]. As a volatile organic compound, benzene is one of the main contributors to air pollutants in the environment. It is found in the environment as a contaminant from both human activities and natural processes. Furthermore, benzene is an intrinsic component of tobacco smoke, and tobacco smokers have a higher body burden of benzene than nonsmokers [6].

Human exposure to benzene has significant deleterious health effects. Exposure to benzene is associated with the risk of blood disorders, including leukemia, lymphoma, aplastic anemia, pancytopenia and chromosomal aberrations [3, 7–10]. In addition, exposure to benzene can cause a wide range of adverse effects on the central nervous system, hematological, hepatic, renal, and lung functions [11–15]. Thus, communities surrounding petroleum refineries have significant health risks due to the increased probability of being exposed to benzene and other toxic chemicals.

Benzene enters the body primarily through inhalation of contaminated air or through direct contact with the skin. It is readily absorbed into the body when inhaled into the lungs and excreted as metabolites such as phenol, benzene oxide, benzoquinone, muconaldehydes, hydroquinone, and catechol in the urine [16]. The toxic effects of benzene are thought to arise from its metabolites, particularly from benzoquinone and the muconaldehydes. Evidence suggests that benzene-induced toxicity involves multiple mechanisms such as oxidative stress, DNA damage, disruption of the cell cycle, and programmed cell death [17–19]. In addition, immune dysfunction has been hypothesized to contribute to benzene toxicity as benzene can interfere with innate, humoral and cellular immunity [20, 21].

In 2010, a flaring incident at the British petroleum (BP) refinery facility led to the release of a huge amount of toxic chemicals into the air in Texas City, Texas [22, 23]. As a consequence, over 500,000 pounds of toxic chemicals, including over 17,000 pounds of benzene was released into the skies threatening the health of the residents of Texas City and the surrounding communities [22–24]. The Galveston County District Clerk’s Office estimates that over 50,000 people are presumed to be affected by the incident.

To understand the potential health effects of ambient benzene exposure resulting from the BP flaring incident, a series of studies were conducted examining the hematological and hepatic functions in affected subjects. The pilot study findings indicated that benzene exposure resulting from the BP flaring incident significantly altered the hematological and hepatic indices in the exposed subjects compared with the unexposed subjects [25]. Assessment of health consequences of benzene exposure in pediatric subjects also indicated similar alterations in hematological and hepatic functions following the same prolonged BP flaring incident in Texas City, Texas [26].

The routine assessment of patients’ hematological profile involving the measurement of their white blood cell (WBC) counts, platelet counts, hemoglobin, hematocrit, blood urea nitrogen (BUN), creatinine and other indices is used to monitor and evaluate the status of a patients’ health including the detection of any changes as a consequence of toxicant exposure, infection, or diseases such as cancer. Similarly, measurement of serum levels of alkaline phosphatase (ALP), aspartate amino transferase (AST), and alanine amino transferase (ALT) are used as markers for hepatic function [27]. Since benzene exposure is linked to the development of multiple hematological malignancies [3, 7–10], we assessed its health effects by examining hematological and hepatic profiles in benzene exposed nonsmoking subjects and compared them with those unexposed nonsmoking subjects.

Since tobacco smokers may have a higher body burden of benzene than nonsmokers, we tried to eliminate this variable by examining the health effects of benzene exposure only among the nonsmoking subjects who were exposed to the prolonged flaring incident that occurred at the BP refinery facility.

## Subjects and methods

### Subjects

**Figure 1:**
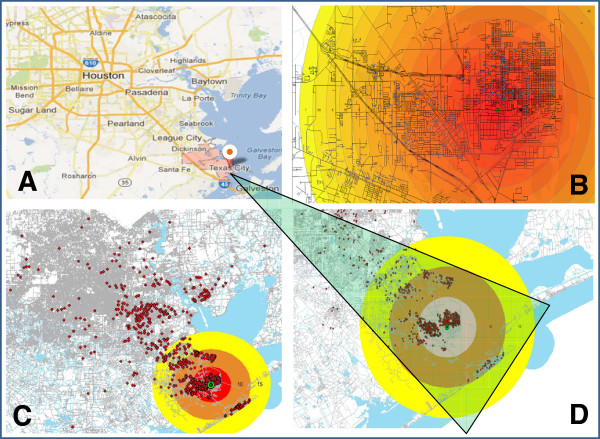
**Map showing the location of the incident of British petroleum (BP) refinery that spewed hundreds of thousands of pounds of toxic chemicals including benzene and carbon monoxide into the skies of Texas City, Texas for 40 days from April 6 to May 16, 2010.** During the incident, the wind originated from south to north and dispersed the discharged benzene impacting the people residing in the northern parts of the Texas City. **A**. Location of Texas City, Texas. **B**. Depicted intensity of benzene exposure from BP incident surrounding neighborhoods of Texas City, Texas. The red, orange, and yellow colors depict the higher (red) to reduced (orange) to low (yellow) intensity of benzene exposure. **C**. Scattered dots represent the location/address of the study participants who were exposed to benzene following a flaring incident at the BP refinery and surrounding areas. **D**. A closer look at the affected area by the benzene exposure and the location of study participants (scattered dots).

This retrospective study was approved by the Quorum Institutional Review Board. The details of the subjects’ selection and the procedures employed for the clinical and laboratory evaluations were reported previously [25, 26]. Briefly, residential areas affected by the BP refinery emission due to the flaring event were initially identified and the subjects exposed to the emission were selected from the affected areas of surrounding communities of Texas City, Texas (Figure 1). Specifically, these subjects experienced a prolonged and involuntary exposure to benzene for up to 40 days following the BP refinery flaring incident that occurred on April 6, 2010 and lasted through May 16, 2010. Subjects who were unexposed to benzene were drawn from primary care clinics located approximately 30–50 miles away from the BP refinery plant [25, 26]. Unexposed subjects had visited the clinic for a routine wellness check up and were selected randomly by the primary care physician. Subjects (exposed and unexposed to benzene) with smoking history were excluded from the study. Demographic and clinical laboratory data were collected and included in this analysis. The study was conducted according to the ethical principles of the Declaration of Helsinki. To comply with the Health Insurance Portability and Accountability Act (HIPAA), confidentiality of information was secured by utilizing text encryption, password protection and limited personnel involvement.

### Data analysis

Medical charts of the benzene exposed and unexposed subjects were reviewed and the clinical data was processed for statistical analysis. Clinical data such as WBC counts, platelet counts, hemoglobin, hematocrit, BUN, creatinine, ALP, AST, and ALT levels were assessed and compared between the benzene exposed and unexposed subjects.

### Statistics

Data from all laboratory examinations in this study was systematically collected from the subjects’ medical charts and subjected to statistical analysis. Descriptive statistics were used to assess patient demographics which included means and standard deviations for each group. Variables included were WBC, platelets, hemoglobin, hematocrit, creatinine, BUN, ALP, AST, and ALT. Student’s *t*-test was used to assess the differences between the benzene exposed and unexposed groups. The significance level was predetermined at an alpha level of 0.05.

## Results

A total of 1422 subjects were included in this study. Of the 1422 subjects, 329 were unexposed and 1093 were exposed to benzene. The subjects’ demographics are shown in Table 1. The mean age of the subjects was 47 years in each group. Among the unexposed subjects (n = 329), there were 131 (40%) male and 198 (60%) female subjects. In the benzene exposed group (n = 1093), there were 610 (56%) male and 483 (44%) female subjects. The median time from the time of disaster to the time of the laboratory testing for the exposed group was 145 (range, 77–439) days.

**Table 1:** **Demographics of the study subjects**

Demographics	Unexposed	Benzene exposed
Total subjects (N)	329 (100%)	1093 (100%)
Mean age	47 years	47 years
Gender		
Male	131 (40%)	610 (56%)
Female	198 (60%)	483 (44%)

The results presented in Table 2 show the differences in hematological and hepatic markers between the unexposed and exposed subjects to benzene. Subjects who were exposed to benzene experienced significantly increased mean WBC counts (× 10^3^ per μL) compared with the unexposed subjects (7.7 ± 2.2 versus 6.8 ± 1.7, P = 0.001). Similarly, the mean platelet counts (× 10^3^ per μL) in the benzene exposed group was significantly elevated compared with the unexposed group (288.8 ± 59.0 versus 245.3 ± 54.4, P = 0.001). The mean hemoglobin (g/dL) levels increased significantly in the benzene exposed group compared with the unexposed group (14.9 ± 1.3 versus 14.0 ± 1.2, P = 0.001). The percentage of hematocrit increased significantly among the benzene exposed subjects compared with the unexposed subjects (43.0 ± 3.0 versus 41.9 ± 3.4, P = 0.001). The mean serum creatinine (mg/dL) levels were significantly increased in the benzene exposed group compared with the unexposed group (1.1 ± 0.4 versus 0.8 ± 0.2, P = 0.001). BUN (mg/dL) levels were significantly increased in benzene exposed subjects compared with the unexposed subjects (15.6 ± 4.6 versus 13.5 ± 3.6, P = 0.001). Compared with the unexposed subjects, benzene exposed subjects had significantly elevated levels of ALP (87.3 ± 22.6 versus 69.6 ± 16.5 IU/L, P = 0.001). The mean AST (IU/L) levels were significantly higher in the benzene exposed group compared with the unexposed group (27.8 ± 6.8 versus 19.1 ± 4.7, P = 0.001). The mean serum ALT (IU/L) levels was increased significantly in the benzene exposed group compared with the unexposed group (33.9 ± 10.1 versus 20.3 ± 8.6, P = 0.001).

**Table 2:** **Comparison of hematological and hepatic indices between unexposed and exposed nonsmoking subjects to benzene**

Variable	Unexposed	Benzene exposed	P Value
	(N = 329)	(N = 1093)	
WBC (× 10^3^ per μL)	6.8 ± 1.7	7.7 ± 2.2	0.001*
Platelets (× 10^3^ per μL)	245.3 ± 54.4	288.8 ± 59.0	0.001*
Hemoglobin (g per dL)	14.0 ± 1.2	14.9 ± 1.3	0.001*
Hematocrit (%)	41.9 ± 3.4	43.0 ± 3.0	0.001*
BUN (mg per dL)	13.5 ± 3.6	15.6 ± 4.6	0.001*
Creatinine (mg per dL)	0.8 ± 0.2	1.1 ± 0.4	0.001*
ALP (IU per L)	69.6 ± 16.5	87.3 ± 22.6	0.001*
AST (IU per L)	19.1 ± 4.7	27.8 ± 6.8	0.001*
ALT (IU per L)	20.3 ± 8.6	33.9 ± 10.1	0.001*

*Differences between benzene exposed and unexposed groups are significant.

*WBC* White blood cells, *BUN* Blood urea nitrogen, *ALP* Alkaline phosphatase*, AST* Aspartate amino transferase, *ALT* Alanine amino transferase.

The findings presented in Table 3 represent the differences in hematological and hepatic markers between the exposed and unexposed subjects to benzene according to the gender. The mean WBC counts (× 10^3^ per μL) were significantly higher in male (7.5 ± 2.5 versus 6.7 ± 1.7, P = 0.001) and female (8.0 ± 1.6 versus 6.8 ± 1.6, P = 0.001) subjects exposed to benzene compared with the unexposed group. Similarly, the mean platelet counts (× 10^3^ per μL) in male (267.0 ± 47.7 versus 233.1 ± 48.7, P = 0.001) and female (316.6 ± 63.5 versus 256.3 ± 53.8, P = 0.001) subjects exposed to benzene was significantly elevated compared with the unexposed group. BUN (mg/dL) was also significantly higher in male (16.1 ± 4.9 versus 14.6 ± 3.2, P = 0.001) and female (14.8 ± 4.5 versus 12.8 ± 3.7, P = 0.001) subjects in the benzene exposed group compared with the unexposed group. Similarly, serum creatinine (mg/dL) levels were also significantly higher in male (1.1 ± 0.3 versus 0.9 ± 0.1, P = 0.001) and female (0.9 ± 0.2 versus 0.7 ± 0.1, P = 0.001) subjects in the benzene exposed group compared with the unexposed group. Conversely, hemoglobin and hematocrit levels did not differ significantly in male or female subjects between the benzene exposed and unexposed groups.

**Table 3:** **Comparison of hematological and hepatic indices between unexposed and benzene exposed nonsmoking subjects according to gender**

Variable	Gender	Unexposed ^δ^	Benzene exposed ^β^	P Value
WBC (× 10^3^ per μL)	Male	6.7 ± 1.7	7.5 ± 2.5	0.001*
	Female	6.8 ± 1.6	8.0 ± 1.6	0.001*
Platelets (× 10^3^ per μL)	Male	233.1 ± 48.7	267.0 ± 47.7	0.001*
	Female	256.3 ± 53.8	316.6 ± 63.5	0.001*
Hemoglobin (g per dL)	Male	15.1 ± 0.9	15.2 ± 0.8	0.13^ψ^
	Female	13.2 ± 0.8	13.5 ± 2.8	0.25^ψ^
Hematocrit (%)	Male	44.9 ± 2.6	44.9 ± 2.2	0.50^ψ^
	Female	39.9 ± 2.3	40.1 ± 2.3	0.10^ψ^
BUN (mg per dL)	Male	14.6 ± 3.2	16.1 ± 4.9	0.001*
	Female	12.8 ± 3.7	14.8 ± 4.5	0.001*
Creatinine (mg per dL)	Male	0.9 ± 0.1	1.1 ± 0.3	0.001*
	Female	0.7 ± 0.1	0.9 ± 0.2	0.001*
ALP (IU per L)	Male	73.7 ± 16.7	84.9 ± 18.3	0.001*
	Female	69.0 ± 18.1	90.3 ± 21.9	0.001*
AST (IU per L)	Male	21.8 ± 5.7	29.9 ± 8.5	0.001*
	Female	18.0 ± 4.4	24.3 ± 6.9	0.001*
ALT (IU per L)	Male	24.3 ± 4.9	40.1 ± 13.7	0.001*
	Female	17.4 ± 6.8	25.3 ± 5.6	0.001*

*Differences between benzene exposed and unexposed groups are significant.

^ψ^ = did not reach statistical significance.

*WBC* White blood cells, *BUN* Blood urea nitrogen, *ALP* Alkaline phosphatase*, AST* Aspartate amino transferase, *ALT* Alanine amino transferase.

^δ^Male unexposed: n = 131; ^δ^Female unexposed: n = 198.

^β^Male exposed: n = 610; ^β^Female exposed: n = 483.

The mean serum ALP (IU/L) levels were significantly elevated in male (84.9 ± 18.3 versus 73.7 ± 16.7, P = 0.001) and female (90.3 ± 21.9 versus 69.0 ± 18.1, P = 0.001) subjects in the benzene exposed group compared with the unexposed group. Similarly, the mean serum AST (IU/L) levels l were significantly higher in male (29.9 ± 8.5 versus 21.8 ± 5.7, P = 0.001) and female (24.3 ± 6.9 versus 18.0 ± 4.4, P = 0.001) subjects in the benzene exposed group compared with the unexposed group. The mean serum levels of ALT (IU/L) was also increased in male (40.1 ± 13.7 versus 24.3 ± 4.9, P = 0.001) and female (25.3 ± 5.6 versus 17.4 ± 6.8, P = 0.001) subjects in the benzene exposed group compared with the unexposed group (Table 3).

To determine if the subjects’ age had any impact on the health effects of benzene exposure, the subjects were categorized into <40 years and, ≥ 40 years age groups and the clinical findings were compared between the unexposed and benzene exposed groups. The results in Table 4 show the differences in hematological and hepatic markers between the exposed and unexposed subjects among the two age groups. Although the mean WBC counts decreased with increasing age in both groups, the mean WBC counts were significantly higher in the benzene exposed age-groups compared with their matched unexposed age-groups. A decreased trend in the mean platelet counts was seen with the increasing age in both groups; significantly higher mean platelet counts were observed in the benzene exposed age-groups compared with their matched unexposed age-groups. Conversely, no significant differences were found in the mean hemoglobin, hematocrit, and BUN levels between the unexposed and benzene exposed subjects irrespective of the age-group. Serum creatinine levels were increased significantly in the benzene exposed subjects compared with those in unexposed subjects, in both age-groups. Similarly, the serum levels of hepatic enzymes (ALP, AST and ALT) were increased significantly in the benzene exposed subjects compared with those of the unexposed subjects, irrespective of age (Table 4).

**Table 4:** **Comparison of hematological and hepatic indices by age group between unexposed and exposed nonsmoking subjects to benzene**

Variable	Age Group	Unexposed ^δ^	Exposed ^β^	P value
WBC (× 10^3^ per μL)	< 40 years	7.3 ± 1.7	8.1 ± 2.8	0.001*
	≥ 40 years	6.6 ± 1.6	7.6 ± 1.8	0.001*
Platelets (× 10^3^ per μL)	< 40 years	254.5 ± 55.3	286.9 ± 69.3	0.001*
	≥ 40 years	239.6 ± 53.1	272.1 ± 71.1	0.001*
Hemoglobin (g per dL)	< 40 years	13.9 ± 1.3	14.1 ± 1.8	0.13^ψ^
	≥ 40 years	14.0 ± 1.2	13.9 ± 1.7	0.13^ψ^
Hematocrit (%)	< 40 years	41.6 ± 3.6	41.8 ± 4.4	0.10^ψ^
	≥ 40 years	42.0 ± 3.3	41.7 ± 4.4	0.13^ψ^
BUN (mg per dL)	< 40 years	12.2 ± 3.1	12.6 ± 5.3	0.27^ψ^
	≥ 40 years	14.8 ± 3.8	15.0 ± 5.7	0.25^ψ^
Creatinine (mg per dL)	< 40 years	0.8 ± 0.2	1.0 ± 0.4	0.001*
	≥ 40 years	0.8 ± 0.2	1.1 ± 0.3	0.001*
ALP (IU per L)	< 40 years	70.2 ± 7.8	84.5 ± 11.3	0.001*
	≥ 40 years	72.6 ± 8.6	89.4 ± 12.2	0.001*
AST (IU per L)	< 40 years	19.1 ± 5.2	24.3 ± 5.7	0.001*
	≥ 40 years	19.8 ± 5.3	26.1 ± 7.2	0.001*
ALT (IU per L)	< 40 years	19.8 ± 6.2	30.7 ± 6.2	0.001*
	≥ 40 years	20.6 ± 6.7	32.1 ± 7.1	0.001*

*Differences between benzene exposed and unexposed groups are significant.

ψ = did not reach statistical significance.

*WBC* White blood cells, *BUN* Blood urea nitrogen, *ALP* Alkaline phosphatase, *AST* Aspartate amino transferase, *ALT* Alanine amino transferase.

^δ^Unexposed < 40 years: n = 128; ^δ^Unexposed ≥40 years: n = 201.

^β^Exposed <40 years: n = 561; ^β^Exposed ≥40 years: n = 532.

## Discussion

Human exposure to benzene is associated with multiple toxicities affecting the hematological, hepatic, immunologic, and chromosomal functions and an increased risk of carcinogenesis. However, the precise mechanism of benzene induced toxic effects is not fully understood. Thus, a thorough understanding of the health consequences of benzene exposure is important for developing approaches to assess the risk in those affected communities. Since tobacco smokers have a higher body burden of benzene than nonsmokers [6], the health consequences of benzene exposure from the BP’s flaring incident may differ among smokers and nonsmokers. Therefore, this study was conducted to investigate the changes in the hematological and hepatic functions only among nonsmoking subjects following their benzene exposure from the prolonged BP’s flaring incident [25]. The clinical outcomes from benzene exposed nonsmoking subjects were compared with those of unexposed nonsmoking subjects.

The findings of the present study indicate that benzene exposure can induce significant alterations in hematological and hepatic functions among nonsmoking subjects. Specifically, mean WBC and platelet counts were significantly increased in benzene exposed subjects compared with those unexposed subjects. Hemoglobin and hematocrit levels were also elevated in benzene exposed subjects compared with the unexposed subjects. Similarly, the BUN and creatinine levels were significantly increased in benzene exposed subjects compared with the unexposed subjects. Although, several previous studies reported similar findings [28–30], their study population included both smoking and nonsmoking subjects.

Previously, it has been shown that benzene and other chemicals present in petroleum refining affecst the liver function [27]. Therefore, we assessed liver function by examining the serum levels of ALP, AST and ALT among benzene exposed subjects and compared them with the unexposed subjects. The results demonstrated that the serum levels of ALP, AST and ALT were found to be elevated in the benzene exposed subjects compared with those unexposed subjects. Several other investigators also reported elevated liver enzymes among subjects exposed to benzene or petroleum products and organic solvents [31–35]. The increased serum levels of these enzymes could be due to the overproduction or release of enzymes from the liver cells in response to stimuli of hepatocellular injury or cell death. However, the exact mechanisms for overproduction or release of these serum enzymes in benzene exposed subjects still remain to be elucidated.

In order to understand the effect of the benzene exposure by gender and age we compared the outcomes by gender as well as by age groups (<40 years and ≥ 40 years). Findings of the study reveal that both the hematological and hepatic functions were significantly affected in the benzene exposure nonsmoking group compared with the unexposed nonsmoking group irrespective of their gender or age.

We acknowledge that this study inevitably has several limitations. A cross-sectional study design allows only hypothesis-generating, and not causality to be investigated. Thus, it is difficult to infer a causality using such a study design because the clinical outcomes were measured at one time point after exposure to benzene. Another shortcoming of this investigation was the retrospective nature of the study. Hence, further verification of the study findings is required through additional prospective randomized studies. However, planning such randomized studies to evaluate the health effects of benzene and other toxic chemical release from a disaster may not be practical.

Nonetheless, the findings of this study reveal that exposure to benzene is associated with significant adverse health effects among nonsmoking subjects. These effects may lead to the impairment in the function of hematological, hepatic, renal and other organ functions. In addition, there is significant scientific evidence that links benzene exposure with an increased risk of carcinogenesis. It is, therefore, important that those who are exposed to benzene be closely followed over time to detect further long-term toxicities to the bone marrow, liver, kidney and other organs affected by the benzene exposure as well as be monitored for the development of secondary malignancies. To achieve this, health care providers need to monitor those benzene exposed individuals with frequent periodic checkups and laboratory blood work. In addition, pulmonary, cardiac, neurologic and other organ functions should be evaluated periodically to monitor the long-term effects of benzene exposure. Furthermore, to fully understand the importance and nature of these effects, longitudinal and mechanistic studies on the health effects of benzene exposure are warranted. As the health impact from benzene exposure is long-lasting, close follow-up studies are necessary to determine its long-term health effects in those affected populations.

## Conclusion

The findings of this study revealed that benzene exposure has a potential to induce both hematological and hepatic alterations among nonsmoking subjects. The hematological alterations include increased WBC counts, platelet counts, and creatinine in benzene exposed subjects compared with unexposed subjects. Increased levels of ALP, AST, and ALT in the serum indicate hepatic injury in subjects exposed to benzene. It is emphasized, however, that this study was not a controlled trial. The observed findings could be influenced by compounding factors that are inherent to the study design and since the procedures used did not follow a predefined scheme, this may have biased the interpretation of the results. Nonetheless, this study has shed light on both the potential short-term and long-term health consequences of benzene exposure among nonsmoking subjects. Additional studies are being conducted to assess the potential health effects in those residents exposed to benzene that resulted from the prolonged flaring incident at the BP refinery facility in Texas City, Texas.
